# Socioecological drivers of water, sanitation, and hygiene (WASH) choices: A qualitative analysis of maternal perspectives in northwest Ecuador

**DOI:** 10.1371/journal.pwat.0000368

**Published:** 2026-01-12

**Authors:** Molly K. Miller-Petrie, Gwenyth O. Lee, Marie L. Spiker, Adriana Lupero, Mauricio Ayovi, William Cevallos, Gabriel Trueba, Joseph N. S. Eisenberg, Karen Levy

**Affiliations:** 1 Department of Epidemiology, University of Washington, Seattle, Washington, United States of America,; 2 Department of Environmental and Occupational Health Sciences, University of Washington, Seattle, Washington, United States of America,; 3 Rutgers Global Health Institute, Rutgers University, New Brunswick, New Jersey, United States of America,; 4 Instituto de Microbiología, Colegio de Ciencias Biológicas y Ambientales, Universidad San Francisco de Quito, Quito, Ecuador,; 5 Centro de Biomedicina, Universidad Central del Ecuador, Quito, Ecuador,; 6 Department of Epidemiology, University of Michigan, Ann Arbor, Michigan, United States of America

## Abstract

Household-level water, sanitation, and hygiene (WASH) interventions do not always achieve sustainable uptake. Research that considers WASH within a socioecological framework, where multi-level factors are interconnected in influencing choices, can inform more effective interventions. To understand WASH preferences and priorities under different socioeconomic and community contexts, we conducted in-depth interviews and freelisting activities with 33 mothers of children under age two participating in the ECoMiD study in northwest Ecuador. Data were inductively coded and connected thematically to the socioecological framework. Select survey data from ECoMiD were analyzed to provide additional context. Maternal WASH choices are driven by factors at each level of the framework. Climatic: seasonal flooding decreases the appeal of WASH investments like cisterns, and household wealth facilitates access in times of climatic stress. Geographic: benefits of WASH access via proximity to piped systems are complicated by quality and consistency concerns, while access from proximity to rivers is complicated by labor requirements. Community: local infrastructure dictates individual options for accessing WASH, and local conditions are dictated by national sociopolitical context and policy decisions. Household: consistent, quality piped water for drinking and chores is the most common maternal WASH preference. WASH choices respond to financial and labor constraints. Individual: mothers value time-savings associated with WASH technologies and access. Maternal decision making operates at the terminus of a chain of broader and interconnected socioecological conditions. The burden of obtaining WASH access is greatest for the poorest households with the least community infrastructure, compounded by seasonal conditions. This contextually grounded study draws attention to how socio-spatial, economic, and environmental constraints interact in the Ecuadorian context to shape lived experience. Improving community-level WASH and taking a multisectoral approach to health interventions would better address barriers to WASH access, and support mothers in making WASH-related choices that can ultimately improve child health and wellbeing.

## Introduction

Recent large-scale household water, sanitation, and hygiene (WASH) interventions have had limited success in interrupting enteric pathogen transmission and improving child health outcomes [1–4]. This may be partially explained by limited acceptability and uptake of externally designed WASH interventions, driven by a lack of understanding of individual and community priorities [5–7]. There is also a need to better understand the upstream factors that impact household WASH access and motivate the prioritization and sustained uptake of new WASH technologies and behaviors. WASH is increasingly recognized as a complex set of interventions managed by diverse authorities [5] that encompass multiple domains, including government policy, climatic conditions, community infrastructure, household hardware, and individual behaviors. Yet less is known about the interplay between these domains – for instance, how individual WASH choices simultaneously relate to socioeconomic resources, community infrastructure contexts, and climatic conditions [8,9]. Although WASH interventions have the potential to improve health, the sector’s focus on improving health outcomes may not always align with the priorities of the people who actually engage in daily WASH decision-making.

WASH research has increasingly recognized non-health motivators for WASH choices, such as privacy, dignity, safety, and quality of life [10,11], as well as the external limitations placed on WASH choice by factors such as gender inequality and poverty [12,13]. Yet global WASH targets are still primarily oriented towards achieving coverage goals (e.g., connection to piped networks) [14], rather than understanding mechanisms for uptake of solutions that can withstand external challenges [15,16]. There have been growing calls to incorporate the perspectives of the individuals who use WASH – particularly women – into water quality and treatment product research [7], as well as to develop alternate WASH service ladders that prioritize service aspects most valued by these individuals [17,18]. More recent research has begun to take a social-justice oriented approach to understanding disparities in water access [19]. By expanding the focus beyond the role of the individual, researchers can better meet community needs that go beyond specific health concerns and consider health and well-being more holistically [17].

Socioecological frameworks consider individual outcomes to be linked to larger societal and structural factors [20,21], and can be particularly useful for understanding motivations for behaviors, including those related to WASH. The identification of specific drivers of WASH-related decision making (“choice”) can help to illuminate the constraints within which individuals act to access WASH resources. Frameworks that incorporate multi-level and cross-cutting barriers and motivators to WASH use in low-resource settings are particularly important for understanding drivers of water choice [22]. Improved understanding can ultimately inform more effective community health interventions.

In Ecuador, government policies that aim to expand rural access to WASH [23], increase maternal empowerment and alleviate poverty [24,25], and strengthen community reliance to climate change [26], outline the political environment within which individuals are able to exercise their WASH choices. Ecuadorian community members themselves are best equipped to identify the specific drivers and constraints of WASH choice that result from this broader context, and community voices can provide important insights on upstream barriers not readily identifiable through quantitative analyses of WASH-related access alone.

In this study, we use qualitative data generated as part of a larger mixed-methods study in northwest Ecuador to understand intersectional drivers of WASH choice across multiple levels of a socioecological framework: climatic, geographic, community, household, and individual. Our overarching objective is to understand socioecological factors that support maternal WASH choices and enable healthy WASH behaviors. Specifically, we aimed to understand the factors that drive maternal choices about which water sources to consume or utilize, and which WASH products and hardware to invest in. Our findings can inform future approaches to WASH interventions.

## Methods

### Study design

This qualitative analysis was conducted in conjunction with an ongoing prospective birth cohort in northwest Ecuador, the ‘Enteropatógenos, Crecimiento, Microbioma, y Diarrea’, or ECoMiD study (in English: enteric pathogens, growth, microbiome, and diarrhea) [27]. Select ECoMiD survey data were included in this analysis to inform the sampling frame and to provide additional context for the results. A socioecological framework, based on the models originally developed by Bronfenbrenner [28] and informed by public health theory put forth by Krieger and others [20], was adapted from the parent ECoMiD study [27] and guided the qualitative study design, analysis, and interpretation of the results.

### Background and study setting

We enrolled 33 interview subjects (all women) from among households already participating in the ECoMiD study [27], between November 6 and November 16, 2023 (see: Data collection: recruitment and interviews). We focused exclusively on mothers to align with the structure of the ECoMiD study and to stay within the boundaries of our existing human subjects approvals.

In the parent study, ECoMiD field workers enrolled 521 mother-child dyads across an urban-rural gradient made up of eight small rural villages, four accessible by road (pops. 500–1,000) and referred to here as “rural-road” communities, and four primarily accessed by river (pops. ~ 200–700) and referred to here as “rural-river” communities. The gradient also includes the mid-sized town of Borbón (pop. ~ 5,000), referred to as “intermediate”, and the larger city of Esmeraldas (~pop. 162,000), referred to as “urban”. Mothers were recruited at the end of their pregnancy and followed until their children turned two; each household was visited 10 times throughout the study. ECoMiD field workers carried out household surveys and spot checks that provided data on WASH conditions (toilet type, water type, handwashing station, water storage containers), socioeconomic conditions (household assets, housing materials, maternal education), and demographic information (sex, age).

The province of Esmeraldas where the study takes place is primarily Afro-Ecuadorian, with a substantial indigenous population, and is among the poorest provinces in Ecuador [29–31]. Most of the communities participating in ECoMiD are located along the Cayapas, Santiago, Onzole, or Esmeraldas rivers, which provide important sources of water, food, and transport. The communities also experience regular exposure to extreme-weather events, such as flooding and landslides due to heavy rainfall that has worsened in recent decades with the changing global climate [32–34].

#### Community infrastructure.

Public piped water systems of varying age, quality, and consistency are present in the urban site, Esmeraldas, the intermediate site, Borbón (water plant constructed in 1990 and upgraded in 2006), and the rural-road communities of Timbiré & Selva Alegre (shared and newer system), Maldonado (older system with poorer perceived quality), and Colon Eloy. A public project to install a piped water system in some of the rural-river communities – Colon de Onzole, Santo Domingo, and Zancudo, but not San Francisco – was under construction but not yet operational as of October 2024. Pipeline supply and pressure vary by proximity to plant and elevation, among other physical factors, and so constraints to use can vary both within and between sites [35,36]. Intermittency of access to piped water is another major driver of variation in access and use across the sites, and different daily and hourly water availability has been well documented in the study area [35,37].

#### Household water access.

Within ECoMiD communities that have public water systems, access to piped systems at the household-level varies. Households in informal settlements (built without government permission, often in flood or landslide prone areas or on otherwise less desirable land that may be difficult to connect to public services), households on the edges of urban areas, and households in rural areas where no piped systems exist are the least likely to have a household connection [35].

#### Community sanitation systems.

A sewer system with a treatment plant is in place in the urban site of Esmeraldas. The intermediate town of Borbón has sewerage pipes that discharge directly into the river. Pipes have also been installed in parts of some communities, such as Maldonado (rural-road), as a part of housing provided by the “Ministerio de Desarrollo Urbano y Vivienda” (MIDUVI), but the drainage pipes are not connected to any larger system. There are no sewer systems in place in the rural-river communities. Households in communities without sewerage systems for processing black wastewater typically rely on septic tanks, soak pits, or pit latrines, and use large buckets stored in the bathroom and shower areas to flush toilets and bathe.

### Data collection: Interview tool development

Each interview included open-ended questions as well as two freelisting activities intended to gather additional information. Open-ended questions covered conceptualizations of wealth and of differences in social classes, difficulties and solutions for accessing WASH, seasonal differences in WASH access, and individual and community priorities, among other related topics. In the first freelist activity, the participants were asked to identify, in any order, the most important objects they or their family needed to 1) get drinking water, 2) get water for chores, and 3) to keep their house clean and hygienic, including feces management (complete interview guide provided in S1 Text). In the second freelist activity, participants were asked to list, in any order, the most important items they owned, of any type. Finally, participants were read and shown an extensive list of WASH-related products, hardware, and technologies and asked which would be priorities that they would want to add to their homes and why. This list of items was designed to match the items included in quantitative survey of WASH access conducted in the parent ECoMiD study.

We pilot-tested the interview-guide with seven fieldworkers across the urban-rural gradient and adjusted in response to their feedback.

### Data collection: Recruitment and interviews

A stratified purposive sample [38,39] was drawn from the ECoMiD cohort, where the unit of analysis was the mother or primary caretaker of a child <2 years of age who was currently participating in ECoMiD, representing the household. To capture maximum variation in the sample, all ECoMiD households were stratified by socioeconomic status (SES) and geographic location on the urban-rural gradient (four sites) to ensure that an equal number of households were included in each strata (see S1 Table), and households were selected based on participant eligibility (child was still <2 at the time of the interview) and strata quotas, as well as based on fieldworker and participant availability on the days interviews were conducted. A total of 33 households across the four sites was considered sufficient to achieve a diversity of perspectives and to have the necessary information power to address the research question [40]. All participants were approached by local Ecuadorian fieldworkers working for ECoMiD for inclusion in the additional interview activity, and asked to sign an additional consent form including consent to audio recording. All participants who were approached agreed to participate, and no repeat interviews were conducted. The final sample consisted of 33 households (9 in the intermediate site and 8 each in the urban site, rural-road site, and rural-river site).

We conducted semi-structured in-depth interviews [41] in Spanish with 33 primary caregivers from November 6–16, 2023 (S2 Table). During each interview, the U.S. researcher (author MKMP) was accompanied by a local ECoMiD project member and/or fieldworker familiar to the mothers. Due to instability in the city of Esmeraldas, we trained two female ECoMiD fieldworkers already based in the city to conduct the interviews for that site. The trained fieldworkers observed MKMP conduct an interview, and each conducted one under observation before commencing their independent interviews. A debrief was conducted at the conclusion of their work. Fieldworkers contacted the participants in advance, and we conducted the interviews during the day in the participants’ homes. Interviews lasted between 30–60 minutes, averaging 45 minutes. We recorded each interview on a small portable recorder, and an Ecuadorian transcribed the interviews verbatim (in Spanish) in December 2023.

A preliminary memo was written by MKMP on positionality and potential pre-conceived notions and biases prior to initiating fieldwork, fieldnote memos were recorded by MKMP at the end of each day of interviews reflecting on the process and findings, and a reflective memo was completed by MKMP at the conclusion of the fieldwork. Memos were incorporated during the analysis stage.

### Data analysis

#### Coding.

After an initial read-through of the complete transcripts, the coder (MKMP) inductively coded [42] text from the interviews in Atlas.ti [43]. Predetermined structural codes related to the socioecological framework, such as “community WASH” and “household WASH” were also utilized. In vivo codes were used if a phrase captured a key shared expression among interviewees best captured in their own words.

The coder (MKMP) maintained a codebook (S3 Table) with a complete description of the definition for each code. Although only one researcher independently coded the data, the codebook and initial categories and themes were shared early on with project team members, including field team members in Ecuador, as a form of peer debriefing and triangulation. Participants were not asked to review their transcripts to avoid placing additional burden on the mothers. Coding was completed in March 2024.

#### Analysis.

We followed recommended practices for thematic analysis [42]: analytic memos were used to identify patterns and categories and to relate the codes and themes with the research questions and the socioecological framework [44,45]. Data processing was conducted primarily through metacoding and cutting and sorting [46] facilitated by Atlas.ti software.

Key methods for pattern identification included comparison of codes and categories between 1) mothers living in different communities along the urban-rural gradient and 2) mothers in different strata of socio-economic status, as determined in the initial sampling frame (S1 Table). Comparison was also made between individual, household, community, geographic, and climatic WASH factors. Findings were presented to and discussed with members of the field team and study team for peer debriefing. After themes were determined, the text of the transcripts was revisited to ensure representativeness and accuracy of the themes as a reflection of the data. Freelists were analyzed using a simple count method to tally the frequency of responses [44,47]. Freelist results were analyzed simultaneously with the open-ended questions so that assessment could be made of divergence or correspondence between the data sources.

### Data protection and ethical approvals

Interview transcripts were stored in a password protected cloud folder hosted by the University of Washington. All data were saved using unique household identifiers, and any names or potentially identifying information were removed from the final dataset. A file linking the household IDs to identifying information was available to the ECoMiD study team and saved in a separate password-protected cloud folder with restricted access.

The ECoMiD study had oversight and approval from institutional review boards at the University of Washington (IRB 00014270) and Universidad San Francisco de Quito (2018–022M), and was also approved by the Ecuadorian Ministry of Health (MSPCURI0002534). The study was originally approved in the United States by Emory University (IRB00101202). All interviews were approved under these IRB protocols. Prior to participating in qualitative interviews, participants signed a consent form, including consent to audio record and store de-identified data, that was separate from their consent to participate in the ECoMiD parent study.

### Analysis team and positionality

The authors acknowledge that our participation in the development of the research question, the undertaking of the research process, and interpretation of findings was influenced by our positionality. For MKMP, this included being a white U.S. doctoral candidate studying WASH and a mother of a child under five, fluent in Spanish but not a native speaker, who has periodically lived throughout Latin America, but never resided in Ecuador. Our partners, collaborators, team members, and study participants in Ecuador provide essential knowledge to inform the research project.

## Results

**Fig 1.**
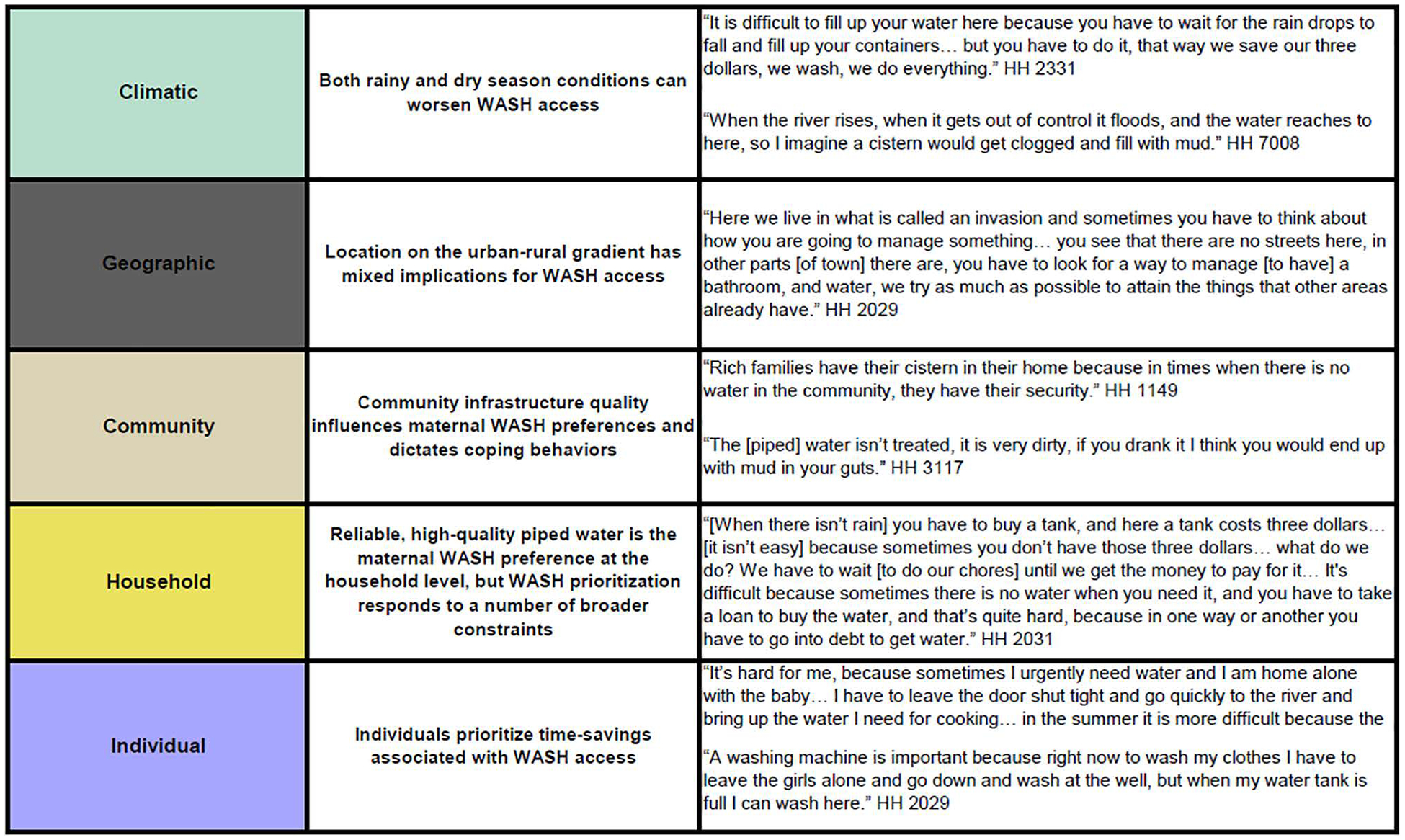
Key findings mapped to a socioecological framework. Levels of the socioecological framework are shown on the left, horizontally linked to key themes identified in the interviews. Quotes illustrate each key theme. WASH = Water, Sanitation, & Hygiene. https://doi.org/10.1371/journal.pwat.0000368.g001

Mothers interviewed ranged in age from 18 to 39, with a mean age of 27 (Table 1). There was over-representation of households in the middle wealth tertile (46%, compared to 24% in the poorest and 30% in the wealthiest). Mothers in the rural river sites were older, on average, and had completed less schooling overall compared to mothers in more urban sites, although this reflects the population in each of these sites. Freelisting results, key themes, and illustrative quotes from the qualitative data analysis are presented below in alignment with the socioecological framework (Fig 1). Original Spanish language versions of all quotes are provided in S2 Text.

### Freelisting results

Overall, purchased bottled water was the most frequently freelisted item as important for obtaining drinking water (70% of interviewees, Table 2), Rainwater, river water, and piped water were the most common items listed as important for getting water for chores. Mothers also discussed purchasing well water from neighbors for this purpose. A number of household water storage container types (e.g., tanks, drums, see S1 Fig) were also listed frequently as important for both drinking water and chores.

There was variation by site: Intermediate site residents most often listed purchased bottled water as important for drinking (89%) (Table 2). The rural-river sites most frequently listed rainwater as important (88%), while in the urban site mothers listed piped water (63%) and purchased water (50%) as important, but not rain. 38% of rural-road interviewees listed piped water as important overall, compared to 75% listing purchased water and 25% listing rainwater.

Rainwater was most frequently freelisted as important for chores in the river communities (listed by 100% of mothers in that site). In the rural-road communities, piped water was most frequently listed as an important source of water for chores (88% overall), while in the intermediate site most households listed rainwater as important for chores (78%), and in the urban site most listed tanks (no specified source, 63%) and pipes (38%).

Chlorine was the most commonly listed item needed for keeping the household clean, including feces management (58% of mothers overall), but was listed less frequently as important for drinking water (12% of mothers overall) or water for chores (6% of mothers overall) (Table 2). Water was the third most frequently listed item as important to keep the household clean (24% of mothers overall), after chlorine and disinfectant.

Cisterns were by far the most common response to household WASH priorities (selected by 66% of mothers overall, ranging from 44% in the intermediate site to 100% of mothers in the urban site), followed by bathrooms (selected by 45% overall, ranging from 13% in the urban site to 67% in the intermediate site). More than 10% of mothers also selected piped water, showers, pumps, and filters or purifiers as priorities to purchase or install.

### Thematic analysis

#### Climatic scale: Both rainy and dry season conditions affect WASH access.

In the dry season, access to water sources becomes more limited, as rivers become smaller and more distant and rains lessen. Many mothers reported having to rely on less-preferred water sources during the dry season, and having to work harder or pay more to access their usual sources.

“It is difficult to fill up your water [containers]… from here, you have to go and fill the buckets, and you get tired, sometimes it doesn’t rain, and there is no money to buy water, it is hard.”

HH 11

“It is difficult when it is the dry season, those who have water, we have to ask them to fill a tank for us, and they charge a dollar fifty for the tank when it doesn’t rain. But when it rains, then even with my [injured] leg I go outside and try to fill my tanks and buckets.”

HH 7008

**Fig 2.**
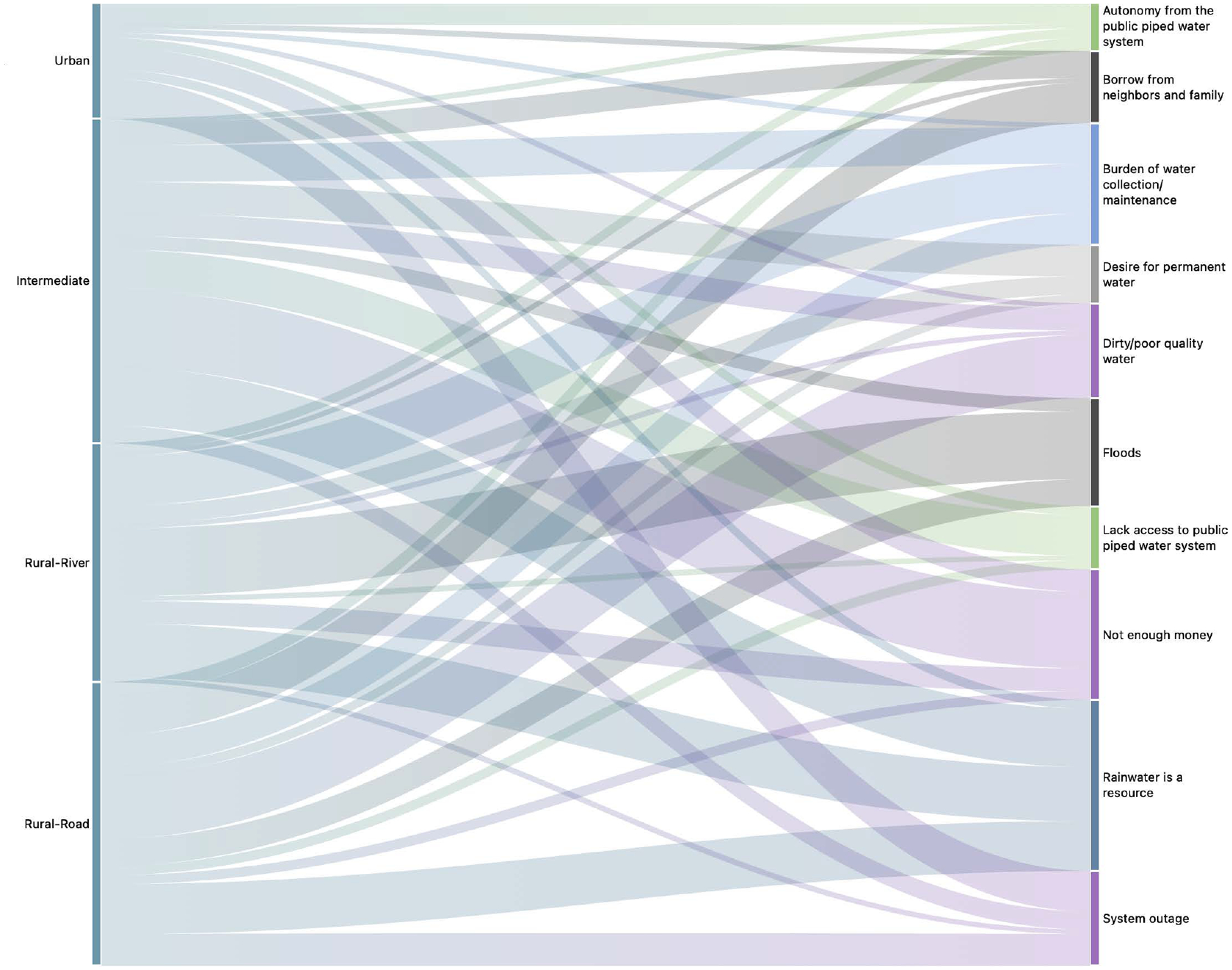
Sankey diagram of select codes by study site. Study sites were located along an urban-rural gradient, and included Esmeraldas (urban site), Borbón (intermediate site), rural sites accessible by road (rural – road), and rural sites accessible only by river (rural – river). Thicker lines represent greater frequency of code occurrence across all interviews from that site. https://doi.org/10.1371/journal.pwat.0000368.g002

However, in the wet season, when rains are more frequent, heavy rains can lead to flooding and cause the rivers in the region to overflow. Many mothers expressed an unwillingness to install expensive household WASH hardware like cisterns in areas like the rural-river communities, where flooding events are common and likely to contaminate stored water. Mothers from rural-river communities talked the most frequently about flooding in the interviews (Fig 2).

#### Geographic scale: Location on the urban-rural gradient has mixed implications for WASH access.

##### Water storage:

Despite the larger extent of public WASH infrastructure available in the more urban locations of the gradient, mothers in the urban site often struggled with water intermittency and expressed a desire for household water storage options like tanks and cisterns to increase reliability of their water supply. Large water tanks and cisterns provide the greatest volume but take up valuable space, while smaller containers like jerry cans and buckets must be refilled often.

##### Drinking water sources:

Even households that have a piped connection to public water do not always report using it as their main source of drinking water. Among the households that participated in the interviews, less than a third reported piped water as their main source of drinking water, while almost half had access to a household piped connection (Table 1).

Bottled water tends to be more expensive when purchased in smaller containers at greater frequency. In the intermediate site, a number of study households were located in informal settlements without access to the public piped system. Many mothers, particularly in the intermediate site and rural-road communities, described the piped water that was available as too dirty to be used for drinking, though piped water was often described as useful for chores (washing floors, clothes, dishes, bathing, cleaning the bathroom, etc.). Mothers in the rural-river communities lacked access to any piped systems, but described easy access to rivers as a benefit.

“Sometimes in the city, you might go without water for three, four days, but here at least from the river we have access to water all the time”

HH 7010

#### Community scale: Infrastructure quality influences maternal WASH preferences and dictates coping behaviors.

The perceived quality of water sources differed across communities within the same geographic area due to infrastructural variability. For instance, the water treatment plants in the rural-road communities of Selva Alegre and Timbiré were newer and tended to have high perceived quality compared to those in Maldonado and Borbón, where the treatment plants were quite old and perceptions of water quality and acceptability [48] were very poor.

“[I would like] the water to come out cleaner, because sometimes it comes out brown, like river water, it comes out dirty.”

HH 2125

Although the ECoMiD study does not collect data on user satisfaction with water, adequate water access has been defined by the AAAQ framework in terms of availability, accessibility, acceptability, and quality, where acceptability is defined by color, small, and taste, and quality is determined by health risk [48]. Availability also varies by community [35], particularly in terms of intermittency of access, with implications for which community members need to consider alternatives and backups during outages to piped systems.

#### Household scale: Reliable, high-quality piped water is the maternal WASH preference at the household level, but WASH source prioritization responds to a number of broader constraints.

Mothers often expressed a desire for access to clean, drinkable water consistently and easily available in their homes for all uses – consumption, cooking, and cleaning. Typically, this access was envisioned as a part of a piped water system.

“[I would like to have] clean water where you can just turn on the hose and see clean water that you don’t have to store… I could use it for the bathroom but also to drink.”

HH 3117

**Fig 3.**
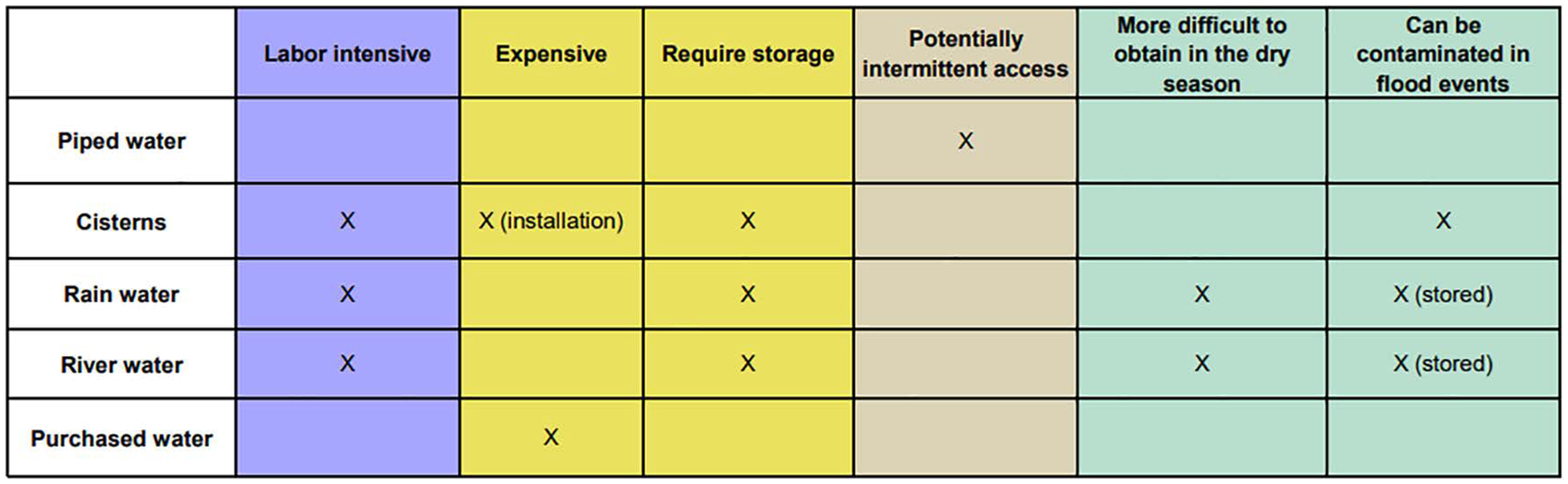
Constraints to maternal water choice, mapped to a socioecological framework. Constraints shown for the most commonly mentioned water sources or storage containers. Shading matches the socioecological levels indicated in Fig 1: Individual level (purple); household level (yellow), community level (brown), climatic level (green). https://doi.org/10.1371/journal.pwat.0000368.g003

In practice, mothers changed their primary water sources based on intended use (drinking vs chores), season, and other conditions, and did not rely on a single source. Mothers described a hierarchy of preferred water sources in response to constraints (Fig 3).

“When it rains hard there are people who collect their rainwater, and when it doesn’t rain, they use their piped water, and only when that is broken and empty, then they go to the river to collect their water, or they go and ask [someone] to fill their water.”

HH 3207

“To get water when it rains, you can collect rainwater, and when you are in a time when it isn’t raining, you have to go get water from the river and boil it, or you have go and buy your bottle of water.”

HH 7008

Water for cooking fell into a middle ground in terms of distinguishing between preferred water sources, with some mothers expressing that “dirtier”/less preferred water sources could still be used in cooking, and others noting that they would only use bottled water for cooking.

“In rich households the water is treated and here in the middle class, for example we have to buy one kind of water to be drinking, and they use the same water for everything because their water is better treated, and ours, if we are talking about the tap water, we can’t use it to cook, we have a specific type of water to cook and another kind of water to do chores or clean.”

HH 2414

Many mothers described cisterns, elevated tanks, or wells connected to a tubing system and pump in the home as valuable when there was no access to piped systems or when that access was unreliable. In the open-ended interview questions, mothers focused primarily on cisterns as a preferred water storage option, and cisterns were mentioned the most frequently as a WASH priority.

“With a cistern you fill it up, and if there is no water you have your water, you don’t go without.”

HH 3207

However, cisterns were considered very expensive and difficult to obtain. In addition, mothers frequently discussed the labor needed to maintain them.

### Storage and labor constraints

Mothers frequently described collecting river and rainwater (and piped water, when systems were inconsistent) and storing it in the household in a variety of containers, from buckets to cisterns (S1 Fig). The labor of collecting river and rainwater, and particularly of transporting river water to the home, was a major focus of many of the interviews. Mothers valued the amount of storage space available in a given storage container because it reduced the frequency with which they needed to collect water. However, once stored, mothers described the burden of keeping the water clean as another time- and energy-consuming activity.

Mothers often mentioned the labor associated with maintaining clean water in cisterns, the top priority WASH item. Cisterns can also pose a risk for arboviral diseases, as they can provide breeding grounds for mosquitos if not properly maintained [49]. Several mothers described the necessity of treating the water using larvicides such as Abate (often distributed for free by the government as a part of dengue prevention efforts) as a part of the associated labor:

“Really, we have to be the ones to treat the water [in the cistern], and to put in the abate and be the ones to be continuously cleaning it, because sometimes the water… cockroaches can get in there… frogs… and sometimes you don’t realize, this water is running all through your house and you think it is well treated, but it isn’t… you have to be cleaning it continuously to make sure you have water security.”

HH 2414

“It would be better if the water came directly from the tap, and I didn’t have to have a cistern, because sometimes the water in the cistern sits for a long time… so you have to be cleaning it, and keep it clean, it isn’t good that the water is like this, it would be better if the water came directly out of the tap.”

HH 2125

Mothers also expressed hesitancy to invest in expensive WASH solutions like cisterns in a house they didn’t own, be it a rental, a family member’s home, or a house in an informal settlement. Mothers also described space or geographic limitations as barriers to constructing cisterns.

“Interviewer: Why don’t you want to build a cistern here?”

“Mother: because it isn’t my house”

HH 3207

“Mother: because there isn’t an adequate place to put it”

HH 4012

“Mother: because the terrain does not lend itself to excavations, it is all pure rock below”

HH 4014

### Financial constraints

When asked about the most difficult part of managing their WASH needs, many mothers expressed that they did not have enough money to manage the amount of water they needed to buy for drinking and for household chores. This issue was discussed most frequently by mothers in the intermediate site (Fig 2). Households that relied on purchased water discussed having to buy large bottles or tanks multiples times a week, and sometimes you just *“don’t have those three dollars”* [HHs 2031, 2331]. Some mothers recognized that buying bottled water might be more affordable in the short term, but was ultimately a less economic solution than buying larger tanks, constructing hardware, or making monthly payments for piped access:

“[Bottled water] might be cheap in the short term, but long term it is expensive buying bottled water, because for example, I buy enough for a month, that makes it easier for me, I don’t have to buy it every day… short term it is cheap but long term it is expensive, if we calculate how much we spend [on water] in a year, but I think bottled water is the most accessible.”

HH 3124

Often, when describing differences between rich and poor households, mothers would describe poor households as needing to be purchasing resources all the time, while rich households “*have everything” (“tengan todo”)* and don’t need to expend time and effort every day to obtain WASH resources.

“They [the wealthy] have more possibilities for getting water, everything we don’t have here, they have everything there, here we have to go and buy and look [for water] every day, but there, they don’t buy [water] every day, they pay monthly, while here we have to pay every day, and when the tank runs out you have to buy another.”

HH 2237

Many mothers expressed that having clean piped water would alleviate many of these daily costs and time burdens, but mothers also described being unable to afford the monthly cost of getting piped water even with a connection. Similarly, WASH items such as cisterns, elevated tanks, and wells were often identified as appealing, but unaffordable.

“If I had it [the money], of course, why wouldn’t I want it [a cistern].”

HH 2031

Mothers also described paying children or other community members to collect water for them, or purchasing water from vendors on the street.

#### Individual scale: Mothers prioritize time-savings associated with WASH access.

Mothers often discussed prioritizing WASH-related purchases in relation to the time-saving benefit that easier access to water or sanitation in the house provided, particularly as a means to free up time for childcare. For instance, household assets such as washing machines were considered valuable in that clothes washing could take place in the home with children present (Fig 1). Similarly, having onsite access to water was often discussed as being valued because it averted the need to leave the home, with or without young children, to collect water. Conversely, many mothers discussed prioritizing purchasing, boiling, or treating water for their children’s consumption, despite the additional cost, time, and labor required to do so.

We also noted some cross-cutting themes that impacted results at multiple scales.

#### Perceived constraints to sanitation access centered on water access.

We included several questions on feces management in the qualitative interviews, but mothers were hesitant to address issues related to feces directly. However, there was a general consensus that water is a necessary tool to enable households to maintain a clean and hygienic environment.

“In order to have a clean and tidy house, you have to have water, if you don’t have water, what else can you do except sweep, how can you clean the bathroom?”

HH 1046

“Water is essential” [repeated by many HHs] – “Water is the most essential in order to keep the house clean”

HH 1149

Most mothers in our study reported owning a toilet that connected to a sewer, septic tank, or pit (Table 1). Few households reported using unimproved sanitation facilities and just two households reported sharing a bathroom with another household. There was a general perception among mothers that household sanitation facilities (primarily septic tanks) were sufficient and without issues – the main issue identified was that water was needed to clean and manage the toilet (e.g., to flush). Mothers did not express concern about their septic tanks filling up or overflowing as long as they could be covered. Some mothers identified sewers as community-level priorities that they would like the government to invest in – but almost always secondarily to playgrounds for children, sports fields, and improvements to roads.

## Discussion

We found that mothers respond to numerous constraints and opportunities at the individual, household, community, and broader geographic and climatic level when making choices about which water sources to consume or utilize, and which WASH products and hardware to invest in (Figs 1, 3). Orienting our analysis around a socioecological framework enabled us to move beyond a narrow focus on individuals and households to consider socioeconomic and environmental drivers of WASH choice at multiple scales, providing insights into how the Ecuadorian context shapes the lived experience of mothers. We found that constraints related to maternal water choice tend to layer together and overlap, particularly in the lower-preference water sources like rainwater and river water, which require labor and storage space and can be more difficult to access or keep clean in both rainy and dry conditions (Fig 3). Higher preference water sources, like piped water and purchased water, had fewer perceived constraints (primarily intermittency and cost, respectively).

Drivers of water choice specifically for chores tended to focus on ease of access over quality, as the water did not need to be fit for consumption. Conversely, the primary drivers of drinking water choice, specifically for children, was quality. Cisterns were frequently mentioned as desirable WASH hardware, but conversely had high financial and labor-related installation costs, in addition to being susceptible to flood events. Coping behaviors, such as purchasing or boiling water, were sometimes discussed as options used to overcome broader constraints (e.g., lack of quality in drinking water), but necessitated additional financial or labor expenditures.

More broadly, mothers experienced the results of political decision-making at the national level through daily management of power outages, flooding, poverty, and varied quality of community water systems, all dictating constantly changing contexts for WASH decision-making.

### Drivers of WASH choice

#### Climatic drivers.

The seasonal impacts of dry and flood conditions dictated maternal WASH choices in different sites in our study (e.g., mothers had to pay for or travel to water during the dry season, and experienced contamination that lessened the appeal of cisterns in flood conditions). In Mexico and elsewhere, year-round water scarce conditions are growing more common, and such scarcity in both access to piped and rainwater has been connected with increased use of WASH coping behaviors [50,51]. Coping mechanisms are generally estimated to be more costly than access to formal infrastructure [52], and so increasing water scarcity is likely to drive increased household WASH expenditures.

Extreme weather events and heavy rainfall are also expected to increase with climate change [53]. Stored water and sanitation systems are both vulnerable to flooding, and while contamination of a stored water source can harm an individual household, contamination from sanitation overflows expands to the community level. Recent research has found that the impact of WASH interventions on health can vary by season [54–56], underscoring the importance of better understanding this driver and providing climate-resilient WASH infrastructure. National level programs that increase climate change resilience, such as those in the Agenda Hábitat Sostenible del Ecuador 2036 [26], can improve the context in which climate-vulnerable communities operate.

#### Geographic and community drivers.

High levels of variation in quality, acceptability, availability, and accessibility to public infrastructure across and within our urban-rural study sites dictated maternal WASH choices. The piped water access provided to households by community infrastructure was particularly important in increasing the ease of access to large quantities of water. Perceived quality of piped water modified mothers’ water choices differently for consumption and for chores. Water used for hygiene has indirect benefits on health [57], and even contaminated or intermittent piped water can provide important reductions in the labor of water collection. In communities without pipes, purchased water, rainwater, and river water were the only options for water access, and purchased water was considered as important for drinking but not for chores in those communities, likely reflecting poverty as a barrier to expenditures and the lesser importance of quality for domestic tasks.

In some instances, there was a clear discrepancy between items that mothers freelisted as household priorities and the opinions expressed in the open-ended interviews. While bottled water for drinking, rainwater for chores, and cisterns for technology were freelisted as the most important household items, in discussion mothers focused on the limitations of these items and expressed a desire for quality public services. These discrepancies indicate that the WASH options currently available to mothers in our study are not meeting their needs.

Intermittency in piped water systems has been found to erode community trust, decrease water security [58,59], and increase reliance on the use of alternate water sources. A recent study in three of the ECoMiD communities found that intermittency increased reliance on bottled water [35,37]. Prior research also found high rates of bottled water purchasing among poor households in the rural-river communities that lack access to any community infrastructure, in place of home treatment methods [37]. Despite the immense household expense and financial concerns voice by the mothers in our study, increasing reliance on bottled water linked to failed public service provision is a trend across low-resource countries [60]. Water testing conducted by our team and others has found that large reusable bottled water sold in Ecuador is contaminated with coliforms, consistent with other global findings [61]. National investment in both rural and urban water systems is essential to improving community level services [23].

Another infrastructural driver of water choice is access to electricity. When the power is out, household technologies that depend on tube and pump systems, drawing water from wells and cisterns, are not able to distribute water throughout the household. The high costs of providing large-scale electrification programs, particularly in rural areas, present a similar challenge as those for constructing large water distribution and sewage systems. Off-grid solutions, such as decentralized grids or smaller, local solar or hydroelectric projects, have been proposed as community-level alternatives for access [62,63]. However, droughts in the region pose a risk for relying on hydroelectric power. Mothers in our study described storing piped and other water in response to planned or predicted outages to cope with these conditions. In 2024, droughts led to widespread electrical outages across South America, including in Ecuador; with climate change, these events are predicted to increase further [64]. Planned power cuts lasting up to 12 hours a day several days a week were commonly taking place throughout the study period of 2022–2024 [64]. Because many WASH systems, like pumps, depend on electricity, cuts to power also mean cuts to water and/or sanitation services in much of the country. WASH solutions provided by governments or via interventions must be able to overcome these infrastructural challenges. There is increasing interest in household-level solutions that can maintain climate resilience in spite of infrastructural deficiencies. However, our results suggest that resources would be better spent on developing more climate-resilient infrastructure at the community level.

Further, infrastructure and electricity limitations are not isolated to individual communities – political will at the national and international level (e.g., SDGs) to provide such services universally, and national capacity to implement such services, dictate the lived reality at the local level. Although we did not ask mothers about sociopolitical context, mothers often brought up these issues, which are a direct result of national processes. Many water systems in smaller communities, including several in our study, are community-financed, with household payments directly supporting system maintenance. There are inconsistent enforcement mechanisms for payment, and non-payment practices may leave such community systems underfinanced, especially without sufficient support and investment from regional and national authorities [65]. Ecuador has experienced increasing sociopolitical instability and violence since 2020 [66,67], with an influx of international narcotrafficking groups and growing levels of corruption in the government, including in the institutions responsible for providing public services such as WASH infrastructure [68]. The province of Esmeraldas already suffers from a lack of investment and maintenance for public projects [69], which is likely to continue under the current context. Historic exclusion of rural, poor, or racialized communities from formal networks during colonization in the region more broadly, as well as the current exclusion of marginalized groups under capitalism, also dictate community level possibilities for water choices [70].

#### Household drivers.

In line with prior research on drivers of water coping practices [51,71–77], labor and costs were major drivers of maternal water choice for both consumption and chores in our study. Water costs included purchasing bottled water, paying someone to collect water, paying for monthly piped water access, paying for fuel or materials to treat water, plus the time and labor costs to collect, store, and treat water from rain or rivers. Both financial and labor expenditures were important and consistent themes expressed by our study participants as burdens that they experienced.

Although our study was limited to female heads of household, mothers are not the only residents making decisions about WASH at the household-level. Women are often more limited in their decision making and in their control over household resources compared to the male head of household [78,79]. In our study population, although some participants reported part-time employment, men were more likely to be the formally employed household member. It is likely that women in Ecuador face similar challenges to exercising agency in financial decision making around WASH as those that have been reported in similar settings elsewhere [78,79], and that gender and gender norms are operating at all levels of the socioecological model.

In addition to gender-based competing priorities for household expenditures, there may also be competing needs for the target of water use at the household level, for example, to attend to agriculture or livestock needs. Both these water-intensive activities can provide important income and nutrition for families, while limiting water availability for other household uses. Both practices can also potentially increase exposure to fecal contamination in the absence of good hygiene and animal and/or crop management practices [80,81].

The percent of household income being spent on water for any use may be extensive for ECoMiD households, given the quantity of water most mothers described needing to purchase on a weekly basis, and the low incomes and relative scarcity of employment options in the study communities. It has been estimated that globally, while a high resource country resident may spend less than 1% of their daily income on water, a low resource country resident could spend nearly 50% of that income on water, and private purchasing [60] can be immensely more costly compared to subsidized access [82]. Programs like Ecuador’s cash transfer (BONO) [25] are important for improving poverty levels overall to enable improved WASH decision making.

Small, daily or weekly water purchases can add up to being much more expensive than monthly connections or larger volume solutions like cisterns, something mothers in our study population were acutely aware of [83]. Mothers in our study area tended to purchase smaller quantities of water from neighbors with wells or piped water connections, mobile water vendors, and from stores, in the absence of being able to connect to a community-level piped system or purchase large volume storage. It is difficult to quantify the exact amount expended by ECoMiD participants using existing survey data, but the burden was clearly expressed in our interviews, and prior research in the study area conducted in 2010 reported monthly water bills for piped connections ranging from less than $3 to more than $30, with frequent reports of non-payment [84]. In our interviews, mothers estimated the monthly cost of piped water at $5-$10 a month depending on volume, and households are charged regardless of outages. One mother mentioned that you could be cut off for non-payment, and that getting the connection reinstated was extremely difficult. The 2010 report also found that more poor households owned a piped connection in areas where government subsidies were provided (Timbiré) compared to in areas without them (Borbón), again highlighting the role of government in community access [84].

Household technology has also been put forward as an additional WASH choice and determinant of subsequent choices [73,85]. In the water realm, technology includes hardware such as cisterns, toilets, water tanks, pumps, and wells that households can purchase or construct. We identified hesitancies in mothers to invest in expensive WASH technologies particularly in houses they did not own, and in informal settlements. Unwillingness to invest in houses in informal settlements is a barrier that was identified as a major constraint to wealth generation in Latin America by Hernando de Soto [86], who promoted legalization of informal settlements as the path towards economic growth and poverty alleviation. In the absence of formal home ownership, local WASH non-governmental organizations in the area have suggested that affordable, temporary alternatives, such as large, transportable plastic tanks, could be a potential solution, but more research with individuals and communities is needed to ascertain if households would want to invest in something more immediately accessible but possibly of perceived lower quality/durability compared to long-term hardware solutions (Personal Comms, Green Empowerment).

#### Individual drivers.

Our findings that mothers make tradeoffs in their WASH decision making, including decisions about utilization, purchasing, and investment, is consistent with existing literature [51,77]. In the context of water choice in low-resource, high-contamination settings, risk of infection is a key part of these tradeoffs [87]. In many instances in the interviews, mothers expressed an awareness that they were making these choices. The frequent discussion of purchasing, treating, or boiling water for young children indicates there is maternal willingness to expend extra time and/or money to achieve perceived health benefits, particularly for this vulnerable age group, but these investments may not fully protect children from infection in this context.

Mothers in our interviews also used social capital to overcome constraints to water access [88], by borrowing water or toilet access from friends, family, and neighbors. Water and toilet borrowing are common coping mechanisms that do not require financial expenditures [88], but which have been connected to increased stress [77]. Water was described by mothers as essential for hygiene in the open-ended interviews, but freelisted less frequently than chlorine and disinfectant. In this case, it is possible that water was viewed as so fundamental a need that it did not always occur to mothers to list it as an “item” they might need, or perhaps the focus on items that decontaminate underscores the connection mothers make between hygiene and health. Either could explain the discrepancy in the findings observed between the methods.

Women tend to be responsible for making WASH-related decisions at the household level [77], while men are more likely to lead infrastructure projects and administer urban water and sanitation systems, ultimately dictating the broader structures of access [89]. This gender disparity has important implications for the limitations placed on women in the WASH process, where they are so often asked to make tradeoffs between costs, labor, and health [77], and their relative inability to alter the broader structural factors at play. If individual preference is rarely considered in designing WASH interventions [17], maternal power and needs may be even less so [7,13,79]. Current efforts need to do more to center the women who bear the brunt of the burden for accessing WASH. A recent review of women’s engagement in WASH interventions found that all interventions included were either gender unequal or unaware [13], meaning that they ultimately did not address the burden on women in providing WASH access for their families. Centering the financial and labor costs of women is likely to lead to more effective WASH interventions. Understanding the various limitations placed on women’s agency at every level of the socioecological model could help direct efforts at empowerment to specific and context-relevant areas of women’s lives.

#### Intersectional drivers.

WASH choices are further dictated by broader contexts that impact each level of the model: Ecuadorian historic, socio-political, economic, and cultural factors, including racialization and gender dynamics, dictate the WASH choices and finances available to communities, decision-making parameters within households, and ultimately the agency of the individuals that live within them. Together, our results and the application of the socioecological analysis suggest that continued and increased public investment in climate resilient infrastructure for rural and urban WASH, poverty alleviation programs focusing on female empowerment, and housing and other multisectoral approaches to health and wellbeing are needed to support individual WASH-related decision making and to improve related health behaviors and outcomes.

Money is a clear enabler of increased options for WASH access and presented households with the means to overcome infrastructural failures and seasonal calamities, among other challenges. Ultimately, household wealth is a downstream outcome of numerous higher-level influences, from historic colonization practices to government investment decisions and national economic resources. Control over a household’s financial resources is also dictated by social and gender norms, as described earlier in this paper.

Lack of access to WASH burdens the poorest households the most – poor households are already less likely to have access to public WASH connections [15], and the poorer quality that access is, the more likely that a household will need to expend additional money or labor to access alternate water sources. The burden of obtaining WASH access globally is highest on the poorest [50,76], and coping costs, financial and labor-related, place the largest burden on the poorest populations and on women [76,90]. Weekly purchasing of water requires both time and money, and increases unpredictability [50] and stress [91], as reflected in our interviews, with likely biological consequences associated with heightened stress, particularly for women.

Installation costs for cisterns, tanks, and other hardware that could maintain access in the face of intermittency or low-quality piped water were high and perceived as insurmountable by many of our study participants. Seasonal droughts and flooding compounded the financial burden on households to purchase or expend extra labor to acquire water, particularly in the rural-river communities without infrastructure. Yet the burden placed on the poorest exists across the urban-rural gradient, due to the variation in infrastructure quality and consistency described above. Infrastructure that is accessible, reliable, and high quality can help reduce the burden on households across wealth levels.

Many mothers suggested that there were limited safe drinking water choices available to even the wealthiest residents in our study sites. As such, it is possible that the majority of the ECoMiD households face similar levels of exposure to unsafe drinking water, but the poor are expending more of their resources to access that water. These expenditures may leave the poorest households more vulnerable to other interconnected challenges of poverty, such as food insecurity and unequal access to electricity, and increase the cumulative risk of poor health outcomes, such as growth shortfalls and stunting. Though the health benefits of improved WASH are clear [57,92–95], our research shows that the choices individuals make in utilizing and investing in WASH are dictated by higher-level factors, including ability to access public infrastructure or to pay for alternate means of accessing water, consistent with other study findings [7,96], and underscores the limitations of assessing WASH access only through a single metric (e.g., a piped connection in the household).

### Connecting drivers of WASH choice to health

The use of multiple drinking water sources is one practice often identified by studies looking at coping strategies – much like households that practice stove and fuel “stacking” of both clean and dirty sources [97], households without access or with limited/intermittent access to clean, sufficient, affordable drinking water are likely to layer less-safe forms of consumption on top of cleaner sources, increasing exposure opportunities [98]. On the other hand, in these intermittent access contexts, households without back-up water sources risk completely losing access to water during outages.

Interventions that aim to address individual preferences may be more successful in disrupting WASH choice constraints [86], preventing the need for water stacking, and ultimately enabling more health-positive behaviors. For instance, more interventions could aim to provide WASH solutions that reduce the time and labor needed to obtain water for chores, which our research indicated are important drivers of WASH choice for mothers. Water for chores is central to sanitation, household cleaning, and personal hygiene activities, all of which translate to indirect health benefits, even if the water itself is contaminated. Given increasing global water and energy shortages, countries are increasingly incorporating water sustainability measures that include recycling and treating wastewater and grey water (water used in sinks and showers) [99]. Frameworks taking a social justice oriented approach to understanding water-related health inequities, such as the Drinking Water Disparities Framework developed by Balazs and Ray [19], could be adapted to low-resource settings to highlight water-related disparities in labor and financial burdens. These data could be used to advocate for expanded access to recycled grey water that could be used for chores.

### Strengths and limitations

There are many limitations to being an “outsider” conducting qualitative research, but we made conscious efforts to mitigate bias that might arise from such a position. The research collaboration between the Ecuadorian site investigators and other project investigators has a history of more than 20 years, and the field team has supported several grant projects implemented in the same project area over a long time span and has trained personnel and served as a source of continual employment for people living in the region for two decades. As such, our research benefits from the expertise of community members and local scientific experts, and field team members accompanied interviewers or conducted interviews themselves, building on their established multi-year relationships with the ECoMiD study mothers.

Although the ECoMiD study has generally recruited a complete or representative sample of mothers in most sites, in Esmeraldas we have been unable to sample the entire population due to safety concerns, which may introduce bias. Our interview sample was purposive rather than random, and we were able to create targeted strata using prior study data and relying on team familiarity in the study site. While we sought to include a diversity of wealth levels, the mothers in the study region tend to be poorer compared to other areas nationally. Although the authors were not able to visit the urban site of Esmeraldas during this research period due to instability, the field workers who did the interviews were residents.

In addition, our interviews only included women, and did not include questions specifically asking about gender roles or dynamics. Men, and the role of men or husbands in household WASH access, were not topics that arose organically in the interviews, which may indicate that men are not greatly engaged in the process, or that such power dynamics effect household decision making so broadly that they did not come to light in our more tightly focused interview questions. Future work could benefit from updated versions of the socioecological model that more explicitly consider the role of gender.

Given that the research team has a long history of engagement with WASH actors in the region, it is possible that mothers might see interviews around WASH products as an opportunity to advocate for themselves or their communities. However, an advocacy-based perspective would be welcome, given the focus of our research question on understanding WASH priorities and needs. To limit this influence, the freelist questions related to objects important for various WASH-related activities were asked at the beginning of the interviews, to avoid introducing bias.

Although transcripts were coded by a single coder, the initial codebook and themes were shared with research team members and field team members before finalizing.

### Future directions

This work highlights several areas of future work. First, community WASH infrastructure interventions should be reevaluated by examining the relative impact of household WASH compared to community WASH infrastructure on child health outcomes [100], and assessing the ways each are impacted by socioeconomic status. This shift is motivated in our work by the numerous barriers to effective household-level WASH solutions and the maternal preference for clean, consistent, piped water in the home. Second, we identified distinct concerns around drinking water quality and water availability for domestic use, suggesting the importance of examining the relative health impacts of mitigating contamination in drinking water compared to improving access to water for chores to illuminate priority investment areas for WASH. Third, given that the province of Esmeraldas is likely to continue to experience extreme weather events [101–103], and the concern registered by mothers in flood prone areas, further information on the seasonality of infections in the region, and how seasonal patterns, behaviors, and preferences may be differentially mediated by wealth, could also inform the development of climate-suitable WASH interventions.

## Conclusions

Mothers, as individuals, operate at the terminus of the socioecological framework, and their ability to make decisions related to their health and wellbeing and that of their children is directly impacted by each of the outer layers, such as by seasonal conditions and existing community infrastructure. In this study we demonstrate that individuals, and particularly mothers, behave in response to constraints that are typically operating at levels outside their control [104].

By listening to individuals and prioritizing the voices and needs of women and the poorest, who currently bear the majority of the WASH burden, the WASH sector may be able to make important progress on delivering more effective, sustainable interventions. Government financing for WASH and other intersectional areas, such as housing, electricity, and poverty alleviation more broadly, is ultimately essential to improve health and wellbeing. By broadening the focus of WASH interventions to be multisectoral, and recognizing the interconnected and indirect benefits of access to individuals, the overall impact of such projects could be greatly increased.

## Supplementary Material

S1 Table. Sampling frame for qualitative interviews

S1 Text: Interview Guide

S2 Text: Original, untranslated Spanish language versions of quotes included in this manuscript, in order of mention

S2 Table. Data overview for in-depth interviews

S3 Table. Codebook.

S1 Fig. Types of household water storage containers or water access points frequently utilized in households in the study region.

**S1 Text. Interview guide.** English and Spanish versions.

(DOCX)

S2 Text. Original, untranslated Spanish language versions of quotes included in this manuscript, in order of mention.

(DOCX)

**S1 Table. Sampling frame for qualitative interviews.** Purposive sample of 33 households selected from the 521 households participating in the ECoMiD study, stratified by socioeconomic status and by location of the household on the urban-rural gradient, including Esmeraldas (urban site), Borbón (intermediate site), rural sites accessible by road (rural – road), and rural sites accessible only by river (rural – river), to ensure a variety of contexts were represented.

(XLSX)

**S2 Table. Data overview for in-depth interviews.** Household ID, date of interview, location of interview, person conducting interview, transcriber, language of interview, and length of interview provided for all interviews conducted as a part of this study.

(XLSX)

**S3 Table. Codebook.** Codes applied to open-ended interview data in Atlas.ti. Category of the code, any clarifying comments, and inclusion and exclusion criteria for applying the code included as applicable. WASH = Water, Sanitation, and Hygiene.

(XLSX)

**S1 Fig. Types of household water storage containers or water access points frequently utilized in households in the study region.** All photos taken by the authors.

(TIF)

## Figures and Tables

**Table 1.** Interview participant characteristics.

	Overall (N = 33)	Urban (N = 8)	Intermediate (N = 9)	Rural - road (N = 8)	Rural - river (N = 8)
**Wealth tertile (asset-based)**					
1 Poorest	8 (24.2%)	1 (12.5%)	3 (33.3%)	3 (37.5%)	1 (12.5%)
2 Middle	15 (45.5%)	6 (75.0%)	4 (44.4%)	1 (12.5%)	4 (50.0%)
3 Wealthiest	10 (30.3%)	1 (12.5%)	2 (22.2%)	4 (50.0%)	3 (37.5%)
**Mother’s age**					
Mean (SD)	27 (6)	28 (7)	22 (3)	26 (5)	32 (6)
**Maximum education level**					
Primary or less	4 (12.1%)	0 (0%)	0 (0%)	2 (25.0%)	2 (25.0%)
Lower secondary	5 (15.2%)	0 (0%)	1 (11.1%)	0 (0%)	4 (50.0%)
Upper secondary	17 (51.5%)	6 (75.0%)	4 (44.4%)	6 (75.0%)	1 (12.5%)
Post-secondary or greater	7 (21.2%)	2 (25.0%)	4 (44.4%)	0 (0%)	1 (12.5%)
**Main source water consumption**					
Bottled water	12 (36.4%)	1 (12.5%)	6 (66.7%)	4 (50.0%)	1 (12.5%)
Piped water connection	9 (27.3%)	4 (50.0%)	1 (11.1%)	4 (50.0%)	0 (0%)
Rainwater	7 (21.2%)	0 (0%)	0 (0%)	0 (0%)	7 (87.5%)
Surface water -river	1 (3.0%)	0 (0%)	1 (11.1%)	0 (0%)	0 (0%)
Tube well	1 (3.0%)	0 (0%)	1 (11.1%)	0 (0%)	0 (0%)
None	1 (3.0%)	1 (12.5%)	0 (0%)	0 (0%)	0 (0%)
Public tap	2 (6.1%)	2 (25.0%)	0 (0%)	0 (0%)	0 (0%)
**Piped connection to house**					
Yes	15 (45.5%)	6 (75.0%)	3 (33.3%)	6 (75.0%)	0 (0%)
**Type of bathroom**					
Toilet - sewer	10 (30.3%)	8 (100%)	1 (11.1%)	1 (12.5%)	0 (0%)
Toilet - septic	11 (33.3%)	0 (0%)	0 (0%)	7 (87.5%)	4 (50.0%)
Toilet - pit	6 (18.2%)	0 (0%)	4 (44.4%)	0 (0%)	2 (25.0%)
Toilet - other place	1 (3.0%)	0 (0%)	0 (0%)	0 (0%)	1 (12.5%)
Pit latrine with slab	2 (6.1%)	0 (0%)	2 (22.2%)	0 (0%)	0 (0%)
Pit latrine without slab	2 (6.1%)	0 (0%)	1 (11.1%)	0 (0%)	1 (12.5%)
Plastic bucket	1 (3.0%)	0 (0%)	1 (11.1%)	0 (0%)	0 (0%)
**Share bathroom**					
Yes	2 (6.1%)	0 (0%)	1 (11.1%)	0 (0%)	1 (12.5%)

Demographic information on the mothers participating in the interviews presented overall and by location of the household along the urban-rural gradient, including Esmeraldas (urban site), Borbón (intermediate site), rural sites accessible by road (rural – road), and rural sites accessible only by river (rural – river).

https://doi.org/10.1371/journal.pwat.0000368.t001

**Table 2.** Frequency of water, sanitation, and hygiene (WASH)-related freelist and priority responses by site.

Items listed	Freq	Rel. Freq	Freq	Rel. Freq	Freq	Rel. Freq	Freq	Rel. Freq	Freq	Rel. Freq
**2a:** Things you need to get the **drinking water** you need for you and your family										
	Overall		Urban		Intermediate		Rural-Road		Rural-River	
Purchased water	23	70%	4	50%	8	89%	6	75%	5	63%
Rain water	12	36%	0	0%	3	33%	2	25%	7	88%
Piped water	9	27%	5	63%	1	11%	3	38%	0	0%
Water tank	8	24%	4	50%	1	11%	1	13%	2	25%
Water drum	7	21%	2	25%	1	11%	0	0%	4	50%
Chlorine treatment	4	12%	1	13%	0	0%	0	0%	3	38%
Cistern	2	6%	2	25%	0	0%	0	0%	0	0%
Well	2	6%	0	0%	1	11%	1	13%	0	0%
Gutters	1	3%	0	0%	0	0%	0	0%	1	13%
Waterfall	1	3%	0	0%	0	0%	0	0%	1	13%
River	1	3%	0	0%	0	0%	0	0%	1	13%
Large water drum	1	3%	1	13%	0	0%	0	0%	0	0%
Bucket	1	3%	0	0%	0	0%	0	0%	1	13%
**2b:** Things you need to get the **water for chores** you need for you and your family										
	Overall		Urban		Intermediate		Rural-Road		Rural-River	
Rain water	16	49%	1	13%	7	78%	3	38%	5	63%
River water	13	39%	2	25%	2	22%	1	13%	8	100%
Piped water	12	36%	3	38%	2	22%	7	88%	0	0%
Water drum	11	33%	2	25%	5	56%	1	13%	3	38%
Water tank	11	33%	5	63%	4	44%	1	13%	1	13%
Well	6	18%	0	0%	5	56%	1	13%	0	0%
Bucket	4	12%	0	0%	0	0%	1	13%	3	38%
Cistern	3	9%	1	13%	1	11%	1	13%	0	0%
Hose	3	9%	0	0%	2	22%	1	13%	0	0%
Large water drum	3	9%	2	25%	1	11%	0	0%	0	0%
Pump	2	6%	0	0%	1	11%	1	13%	0	0%
Chlorine treatment	2	6%	1	13%	0	0%	0	0%	1	13%
Gutters	1	3%	0	0%	0	0%	1	13%	0	0%
Tubes	1	3%	0	0%	0	0%	1	13%	0	0%
Tap	1	3%	0	0%	0	0%	1	13%	0	0%
Tank truck	1	3%	1	13%	0	0%	0	0%	0	0%
Items listed	Freq	Rel. Freq	Freq	Rel. Freq	Freq	Rel. Freq	Freq	Rel. Freq	Freq	Rel. Freq
**2c:** Things you need to keep your house **clean and hygieni**c, including feces management										
	Overall		Urban		Intermediate		Rural-Road		Rural-River	
Chlorine treatment	19	58%	3	38%	5	56%	4	50%	7	88%
Disinfectant	12	36%	3	38%	1	11%	3	38%	5	63%
Water	8	24%	5	63%	2	22%	1	13%	0	0%
Soap	8	24%	2	25%	1	11%	3	38%	2	25%
Broom	4	12%	2	25%	1	11%	0	0%	1	13%
Mop	4	12%	2	25%	2	22%	0	0%	0	0%
**2d: Priorities** to add to your home related to **water or sanitation** (keeping your house clean and hygienic)										
	Overall		Urban		Intermediate		Rural-Road		Rural-River	
Cistern	20	66%	8	100%	4	44%	4	50%	4	50%
Bathroom	15	45%	1	13%	6	67%	4	50%	4	50%
Piped water	11	33%	2	25%	3	33%	3	38%	3	38%
Shower	7	21%	2	25%	1	11%	1	13%	3	38%
Clean, uncontaminated, or treated water	7	21%	3	38%	2	22%	0	0%	2	25%
Elevated tank	6	18%	1	13%	0	0%	2	25%	3	38%
Pump	6	18%	3	38%	1	11%	1	13%	1	13%
Filter or purifier	4	12%	2	25%	0	0%	1	13%	1	13%
Sewer system	3	9%	1	13%	2	22%	0	0%	0	0%
Tubing	3	9%	1	13%	0	0%	2	25%	0	0%
Permanent or consistent access to water	3	9%	1	13%	2	22%	0	0%	0	0%
Washing machine	2	6%	0	0%	0	0%	2	25%	0	0%
Tap	2	6%	0	0%	0	0%	0	0%	2	25%
Hose	2	6%	0	0%	1	11%	1	13%	0	0%
Well	2	6%	0	0%	0	0%	1	13%	1	13%
Bottled water	2	6%	0	0%	1	11%	1	13%	0	0%
Sufficient water	1	3%	1	13%	0	0%	0	0%	0	0%
Dishwasher	1	3%	0	0%	0	0%	0	0%	1	13%
Counter you can clean	1	3%	1	13%	0	0%	0	0%	0	0%
Cement bathroom floor	1	3%	0	0%	0	0%	1	13%	0	0%
Large water drum	1	3%	0	0%	1	11%	0	0%	0	0%
Big water tank	1	3%	0	0%	0	0%	0	0%	1	13%

Results are shown beneath each WASH topic. The three freelists are in response to the prompt “please list, in any order, the things you and your family need to…”: 2a get the drinking water you need; 2b get water for chores; 2c keep your house clean and hygienic; 2d prioritize to add to the home related to WASH. WASH priorities were determined by selection from a pre-set list or direct mention during open-ended interviews. Blue shading highlights the frequency of responses, with darker blue indicating higher frequencies either overall or separately by location of the household along the urban-rural gradient, including Esmeraldas (urban site), Borbón (intermediate site), rural sites accessible by road (rural – road), and rural sites accessible only by river (rural – river).

https://doi.org/10.1371/journal.pwat.0000368.t002

## Data Availability

The interview guide and codebook are provided in the Supporting information.
