# Supplementary material for: Socioecological drivers of water, sanitation, and hygiene (WASH) choices: A qualitative analysis of maternal perspectives in northwest Ecuador
Source: PLOS Water. Author manuscript; Available in PMC 2026 Feb 28. (PMC12948185; doi:10.1371/journal.pwat.0000368)
Supplement: S1 Text: Interview Guide [file NIHMS2143457-supplement-S1_Text__Interview_Guide.docx]

**S1 Text: Interview Guide.** English and Spanish versions.

Interview guide

Thank you for participating in this interview. The purpose of this activity is to understand more about your opinions – there are no right or wrong answers. I am here to listen and to understand more about you and your family, and I hope that I can use what I learn to improve public health projects in communities like this one. The interview will last about an hour. *[Read the informed consent form, obtain consent and signature for the interview and ask permission to record the audio]*

**PART 1: ASSETS**

We will start with an activity in which I will ask you to make a list of objects. You can name all the objects you think of, with no limit.

- Please list the most important things that your household owns *[Freelist – please record items in the exact order they are listed]*
  - *[Prompts: can you think of any other things? Look around the room/your house/your yard, do you see anything else you want to add to the list? Can you point to or show me anything else you own that should be on the list? Can you think of any other things like item Y (first item mentioned, second, third) to add to the list?]*
  - Follow up: Why did you choose these *[top]* items? Why are they important to you?
- Picture a wealthy household in your community. What do they own?
  - *[Prompts: Can you think of anything else? What would be in their home? Their yard? What would they buy? What would they do? Can you think of anything else similar to item Y?]*
  - Follow up: Is there anything they do that you feel like you cannot do?
- What do you feel that you can’t do because of a lack of money? Are there things that are important to you that you are not able to spend money on currently, but wish you could?
  - For example, education, travel, health, cultural activities, family time, exercise?
- If you had $1,000 to spend on your household, what would you spend it on? Why?

Pile sort 1: What items are the most symbolic of household wealth? Please order/classify these cards into groups that everyone, from the poorest household and up could own, the middle class and up could own, or that only the wealthiest class would own. There are no correct or incorrect answers, we are only interested in what you think.

You can make as many piles as you want, but please don’t put all the cards into one pile. For instance, please put all the things only a wealthy household would own together. How do you know they are wealthy? If you think important things are missing, we can add cards *[if the participant mentioned something in the freelist that is not on a card, add it to the pile sort].*

Cards:

| Radio | DVD Player |
| --- | --- |
| Non-mobile phone | Plot of Land |
| Microwave | Chainsaw |
| Television | Motor |
| Refrigerator | Bank account |
| Computer | Washing Machine |
| Wristwatch | Air conditioner |
| Internet | Shower |
| Mobile phone | Hot Plate |
| Bicycle | Iron |
| Motorcycle | Oven |
| Scooter | Fan |
| Carriage pulled by animals | Video Camera |
| Car or truck | Exercise Machine |
| Boat with an engine | Sandwich maker |
| Blender | Electric Mixer |
| Gas Stove | House |
| Sewing Machine | Electricity |
| Stereo | Generator |
| Canoe | Livestock |

- Is it important how many of each item a family has? Why and for which things?
- If you could only choose two cards to represent each group, which would they be?
- Which items are the most important to have in your house, in your opinion? Why?
- Please describe a house owned by someone in the upper class/richest.
- Please describe a house owned by someone in the middle class.
- Please describe a house owned by someone in the lower class/poorest.

*[At the end of the activity, list each pile’s “name” and list the number associated with each card under that name]*

**PART 2: WASH**

In this part of the conversation, I would like to learn more about how you use various things in your house and in your daily life, and which things are most important. In particular, I am interested in how you and people in your community get the water you need to drink and do chores, and how you keep your households clean, including managing the feces of your family and animals. First, I am going to ask you to make three lists of objects. They are all similar, but focus on a different topic.

- What are the most important things that your household owns that help you and your family to have the drinking water that you need? *[Freelist – please record items in the exact order they are listed]*
  - *[Prompts: Can you think of anything else you use? Can you look in your kitchen, yard, or bathroom and think about what items might be there that you use? Can you think of anything else similar to item Y you might want to add?]*
  - Can you tell me about why you picked these things? If you don’t already own them, why not? What would it take for you to decide to purchase/build them?
  - Do these things change from season to season, during flooding or droughts?
- What are the most important things that your household owns that help you and your family to have the domestic water/water for chores that you need? *[Freelist – please record items in the exact order they are listed]*
  - *[Prompts: Can you think of anything else you use? Can you look in your kitchen, yard, or bathroom and think about what items might be there that you use? Can you think of anything else similar to item Y you might want to add?]*
  - Can you tell me about why you picked these things? If you don’t already own them, why not? What would it take for you to decide to purchase/build them?
  - Do these things change from season to season, during flooding or droughts?
- What are the most important things that your household owns that help you and your family to keep your house clean and hygienic, including managing feces of people and animals? *[Freelist – please record items in the exact order they are listed]*
  - *[Prompts: Can you think of anything else you use? Can you look in your kitchen, yard, or bathroom and think about what items might be there that you use? Can you think of anything else similar to item Y you might want to add?]*
  - Can you tell me about why you picked these things? If you don’t already own them, why not? What would it take for you to decide to purchase/build them?
  - Do these things change from season to season, during flooding or droughts?
- Picture a wealthy household in your community.
  - What do they have to get the drinking water they need? Are there differences for them in different seasons?
  - What do they have to get the domestic water/water for chores they need?
  - How do they keep their household hygienic/clean?
  - What are the most important differences between a wealthy household and a poor household in your community?

Pile sort 2: Please order these cards in order of priority in which you would want to add them to your house to help with having drinking water, water for chores, and to maintain a clean and hygienic house, if you could add anything without any cost to you. For example, if you could add, construct, or buy any of these items for your house, which would you buy first? If you think we are missing important objects, we can add cards.

Cards:

| Well | Stored water |
| --- | --- |
| Spring | Cistern |
| River | Pomas |
| Piped water connection | Tanks/Water drum |
| Public tap | Water treatment-chlorine |
| Small tank truck/drum | Water filter |
| Rain water | Diapers |
| Bottled water | Toilet discharging to sewer or septic tank |
| Access to water in the house | Pit latrine |
| Access to a neighbor’s water | Composting toilet |
| Quality of drinking water (is good quality) | Cement bathroom floor |
| Color of drinking water (looks good) | Access to bathroom in the house |
| Taste of drinking water (tastes good) | Access to a neighbor’s bathroom |
| Consistent access to water (always available) | Sink |
| Reliable access to water (knowing when it will come) | Soap |
| Washing machine | Shower |
| Mop/broom | Refrigerator |
| Kitchen counter that can be wiped clean |  |

- If you had access to piped water, what would you next priority be? Why?
- Are there items on these cards you don’t want to have? Why?
- Which of these items are the most difficult to obtain? Why? Which are the most expensive?
- What are the biggest differences between how a wealth and a poor house use these items?

*[At the end of the activity, list the number associated with each card under each wealth category*

Open ended questions

- What do you think the most difficult part of managing your household’s water is? What is the easiest?
  - What would help you get the water you need?
  - Are there things you don’t spend money on currently that you think are important to manage your household’s needs and wish you could?
  - What items related to water are daily purchases and which are bigger, long-term purchases?
  - What things could you build to help with this process?
  - What chores are related to water?
- What do you think the most difficult part of keeping your house clean and hygienic is, including managing feces? What is the easiest?
  - What type of toilet do you have? How do you dispose of its contents? Is there a water treatment plant in your community? What do you do when there is no water?
  - What would help you maintain a clean and hygienic house?
  - Are there things you don’t spend money on currently that you think are important to manage your household’s needs and wish you could?
  - What items related to hygiene are daily purchases and which are bigger, long-term purchases?
  - What things could you build to help with this process?
  - What chores are related to hygiene?
- If you had $1,000 to spend on your household but could only spend it to get drinking or domestic water, use the bathroom, or keep your household hygienic, what would you buy or build? Why?
- How much money would you need to have available in order to build a new toilet or build a cistern or tank? Why would you or would you not want to construct or invest in these items?
- Do you feel like the community you live in makes a difference in your ability to get drinking or domestic water for your household, use the bathroom, or keep your household hygienic?
  - Is there a functioning sewer system in your community?
  - How so or why not? Do mothers in other communities have an easier or harder time getting the drinking or domestic water they need for their households, using the bathroom, and keeping their households hygienic? How/why?
  - What do you wish you had in your community that you don’t currently have?
  - What would your ideal community have?
- What do you do to avoid getting diarrhea?
  - Does any of it depend on where you live, what you can buy or on how much money you have?
  - What do mothers in other communities do to avoid getting diarrhea?
- What do you do to avoid getting dengue?
  - Does any of it depend on where you live, what you can buy or on how much money you have?
  - What do mothers in other communities do to avoid getting dengue?

***Guia de entrevista***

Gracias por su participación en esta actividad. El propósito de este ejercicio es entender más sobre sus opiniones – no hay respuestas correctas, ni incorrectas. Estoy aquí para escuchar y aprender más sobre usted y su familia, y espero poder utilizar lo que aprendo para mejorar proyectos de salud público en comunidades como esta. La entrevista durara más o menos una hora. *[Hacer el proceso de consentimiento con la forma oficial, pedir permiso para grabar el audio]*

**PARTE 1: LOS BIENES/LAS POSESIONES**

Empezamos con una actividad en que voy a pedir que usted haga una lista de objetos. Puedes nombrar a todos los objetos que piense usted, sin límites.

- Por favor, dígame cuales son las cosas/bienes/posesiones **materiales** más importantes que tiene su familia/su casa. *[LISTA – por favor, registre las posesiones en el orden mencionado]*
  - *[¿Puede pensar en alguna otra cosa? ¿Mira alrededor de su casa, su patio, hay alguna otra cosa que quiere añadir a la lista? ¿Puede señalar o muéstrame alguna otra costa que debemos incluir en la lista? ¿Puede pensar en alguna otra cosa parecida a XXXX para incluir en la lista?]*
  - ¿Porque eligió estas cosas? ¿Porque son importantes a su familia?
- Imagínese una familia rica en su comunidad. ¿Qué tiene esa familia/casa?
  - *[¿Puede pensar en alguna otra cosa? ¿Que estaría en su casa/hogar? ¿Su patio? ¿Qué comprarían ellos? ¿Qué harían ellos?]*
  - ¿Hay algo que ellos pueden hacer, que usted y su familia no puedan?
- ¿Hay cosas que sienta usted y su familia que no puedan hacer por falta de dinero?
- ¿Hay cosas que son Importantes para usted y su familia en las que no pueden gastar dinero en este momento, pero le gustaría hacer?
  - Por ejemplo, ¿viajar, su salud, educación, actividades culturales, tiempo con la familia?
- Si usted tuviera $1,000 dólares para gastar (para usted/su familia/su casa), ¿cómo lo gastaría? ¿Porque?

Sortea de pilas 1: ¿Cuáles posesiones son las más importantes como señales de la riqueza de una familia? Por favor, ordena/clasifique estas tarjetas en **grupos de lo que tendría una casa de clase alta, una casa de clase media, y una casa de clase baja.** No hay respuestas correctas ni incorrectas, estoy muy interesada en que piensa usted.

Usted puede hacer tantas pilas/categorías/montones como quiera, pero no pueda poner todas las tarjetas juntas en una sola pila/montón. Por ejemplo, ponga cosas que indica/significa que una casa es de clase alta juntos. ¿Cómo sabe que la casa tiene mucho dinero? Si parece que falta unas cosas importantes, podemos añadir tarjetas. *[si la participante ha mencionado unas cosas nuevas en la actividad de lista, añade tarjetas con estas cosas al sorteo].*

Tarjetas:

| Un Radio | Un Reproductor de DVD |
| --- | --- |
| Un Teléfono no móvil | Un Solar |
| Una Microonda | Una Motosierra |
| Un Televisor | Un Motor |
| Un Refrigerador/nevera | Una Cuenta bancaria |
| Una Computadora | Una Lavadora |
| Un Reloj de pulsera | Aire acondicionado |
| El Internet | Una ducha |
| Un Teléfono móvil | Un calientaplatos |
| Una Bicicleta | Una plancha |
| Una Motocicleta | Un horno |
| Una Pasola/Scooter | Un ventilador |
| Un Carro tirado por animales | Una cámara de video/videocámara |
| Un Coche o un camión | Un aparato de ejercicio |
| Un Barco con un motor | Una sandwichera |
| Una Licuadora | Una batidora eléctrica |
| Una Estufa de gas | Una casa (dueño) |
| Una Machina de coser | La electricidad / la energía |
| Un Estereo | Piso de cemento |
| Una Canoa | Ganado/animales de la hacienda |
| Paredes de cemento |  |
| Techo de metal |  |
| Techo tejada |  |

- ¿Es importante el número total de las cosas que tiene la familia? ¿Para cuales posesiones?
- ¿Si solo podría elegir **dos** de las tarjetas para representar cada grupo, cuáles serían?
- ¿Cuáles posesiones son lo más importantes a tener en su casa, específicamente, en su opinión? ¿Porque?
- Por favor, describe la casa de clase alta ¿Qué cosas usted ha incluido? ¿Porque? ¿Cuáles son los más importantes?
- Por favor, describe la casa de clase medio. ¿Qué cosas usted ha incluido? ¿Porque? ¿Cuáles son los más importantes?
- Por favor, describe la casa de clase baja. ¿Qué cosas usted ha incluido? ¿Porque? ¿Cuáles son los más importantes?

*[Después de completar la actividad, registra el “nombre” de cada pila y los números de las tarjetas en la pila]*

**PART 2: WASH**

En esta parte de la conversación, quiero aprender más sobre como utilizan varias cosas en su casa y en su vida diaria y cuales cosas son lo más importantes. En particular, estoy interesada en cómo la gente en su comunidad y usted obtengan agua para beber, como obtengan agua para uso en la casa/para oficios, y como mantienen una casa limpia e higiénica, incluso de como manejan las heces de la familia/sus animales. Primero voy a pedir que haga tres listas de objetos. Son todos parecidos, pero cada lista tiene un enfoque/tema un poco diferente.

- Por favor, dígame cuales son las cosas más importantes que tiene su familia/una casa en su comunidad para tener el agua para consumo/para beber que necesitan. *[LISTA – por favor, registre las posesiones en el orden mencionado]*
  - *[¿Puede pensar en alguna otra cosa que utilizan? ¿Puede mirar en su cocina, su baño, o su patio, y pensar en que tipos de objetos utilizan allí? ¿Puede pensar en alguna cosa parecida a XXXX para incluir en la lista?]*
  - ¿Me puede explicar porque has pensado en estas cosas?
  - ¿Hay algunos en la lista que usted no tenga? ¿Porque no ha comprado o construido XXX cosa?
  - ¿Las cosas importantes cambian entre la temporada de lluvia y estación seca, con inundaciones, o con sequias?
- Por favor, dígame cuales son las cosas más importantes que tiene su familia/ una casa en su comunidad para tener el agua domestica/para oficios que necesitan. *[LISTA – por favor, registre las posesiones en el orden mencionado]*
  - *[¿Puede mirar en su cocina, su baño, o su patio, y pensar en que tipos de objetos utilizan allí? ¿Puede pensar en alguna cosa parecida a XXXX para incluir en la lista?]*
  - ¿Me puede explicar porque has pensado en estas cosas?
  - ¿Hay algunos en la lista que usted no tenga? ¿Porque no ha comprado o construido XXX cosa?
  - ¿Las cosas importantes cambian entre la temporada de lluvia y estación seca, con inundaciones, o con sequias?
- Por favor, dígame cuales son las cosas más importantes que tiene su familia/ una casa en su comunidad para mantener una casa limpia e higiénica - incluso el manejo de las heces de las personas, los niños, y los animales. *[LISTA – por favor, registre las posesiones en el orden mencionado]*
  - *[¿Puede pensar en alguna otra cosa que utilizan? ¿Puede mirar en su cocina, su baño, o su patio, y pensar en que tipos de objetos utilizan allí? ¿Puede pensar en alguna cosa parecida a XXXX para incluir en la lista?]*
  - ¿Me puede explicar porque has pensado en estas cosas?
  - ¿Hay algunos en la lista que usted no tenga? ¿Porque no ha comprado o construido XXX cosa?
  - ¿Las cosas importantes cambian entre la temporada de lluvia y estación seca, con inundaciones, o con sequias?
- Imagínese una familia rica en su comunidad. ¿Qué tiene esa familia?
  - ¿Que tienen para obtener el agua para consumir que necesitan? ¿Hay diferencias para ellos entre la temporada de lluvia y estación seca, con inundaciones, o con sequias?
  - ¿Que tienen para mantener una casa limpia e higiénica? - Incluso el manejo de las heces de las personas, los niños, y los animales.
  - ¿Cuáles son las diferencias más importantes entre como una casa de clase alta y uno de clase baja manejan estas cosas?

Sortea de pilas 2: **Por favor, ordenar estas tarjetas en el orden de la prioridad de tenerles en su casa para ayudarle tener agua suficiente a beber, para sus tareas/oficios, y para mantener una casa limpia/higiénica, si podría añadirles horita sin costo.** ¿Por ejemplo, si usted podría añadirles, construirles, o tenerles en su casa, cuales quería poner primero? Si parece que falta unas cosas importantes, podemos añadir tarjetas.

ALTERNATIVA: Pregunta abierta: **Cuales cosas usted considera prioridades a poner en su casa para ayudarle tener agua suficiente a beber, para sus tareas/oficios, y para mantener una casa limpia/higiénica, ¿si podría añadirles horita sin costo?** ¿Por ejemplo, si usted podría añadirles, construirles, o tenerles en su casa ahora, cuales quería poner primero?

- ¿Si usted tenía acceso consistente a agua de grifa, hay otras cosas que serían prioridades? ¿Cuales? ¿Porque?
- ¿Hay cosas en las tarjetas que quieres tener? ¿Porque?
  - ¿Que no quieres tener por razón de la falta de agua?
- Cuales cosas son las más caras/ difícil obtener? Y los más baratos/fáciles de obtener?

Tarjetas:

| Pozo con tubería/protegido | Motobomba |
| --- | --- |
| Manantial protegido | Agua guardada y cubierto |
| Agua superficial - río | Cisterna |
| Agua potable | Pomas |
| Grifo público | Tanque /tambor |
| Carro-tanque pequeño/tambor o Camión cisterna | Agua tratado con cloro |
| Agua lluvia | Agua tratado con abate/abate |
| Agua embotellada | Filtro de agua |
| Acceso a agua dentro del hogar | Inodoro que descarga al sistema de alcantarillado o tanque séptico |
| Acceso a agua de un vecino | Letrina de pozo mejorada y ventilada |
| Un Fregadero/Lavamanos | Panales |
| Agua de buen color | Piso del baño de cemento |
| Agua de buen sabor | La letrina o inodoro adentro de la casa |
| Acceso consistente al agua | Acceso a una letrina o un inodoro no compartida |
| Agua sin contaminación | Acceso a una letrina o un inodoro de un vecino |
| Una lavadora | Jabón en la casa |
| Una fregona/escoba | Ducha en la casa |
| Un mostrador de cocina que puede limpiar | Una refrigeradora/nevera |

- ¿Si usted tenía acceso consistente a agua de grifa, hay otras cosas que serían prioridades? ¿Cuales? ¿Por qué?
- ¿Hay cosas en las tarjetas que no quieres tener? ¿Por qué?
  - ¿Que no quieres tener por razón de la falta de agua?
- ¿Porque son tan importantes las cosas que son sus prioridades? ¿Que facilitan? ¿Sin estos, que no puede hacer?
- ¿Por qué no es importante tener XXX (ejemplo de una cosa no elegida como prioridad)? ¿Si ninguna de esas cosas es importante a tener, cuales cosas serían importantes?
- ¿Hay algunas cosas que no funcionan o no valen la pena tener si no tienen otra cosa para facilitar su uso?
- ¿Cuáles cosas usted quiere tener en su propia casa? ¿Hay otras cosas no en las tarjetas que le gustaría tener en la casa?
- Cuales cosas son las más caras/ difícil obtener? Y los más baratos/fáciles de obtener?
- Cuáles serían las diferencias más significantes/importantes entre una casa de clase alta y una casa de clase baja, en términos de tener y utilizar estas cosas?

*[Después de completar la actividad, registra el “nombre” de cada pila y los números de las tarjetas en la pila]*

Preguntas abiertas

- ¿Qué piensa usted es la parte más difícil de manejar/gestionar las necesidades de su y su familia de agua? ¿Qué parte es lo más fácil?
  - ¿Que ayudara a usted a tener el agua que necesita?
  - ¿Hay cosas en que no gasta dinero en este momento, pero que piensa que son importante para sus necesidades y compraría si podría?
  - ¿Cuáles cosas relacionadas al agua son compras diarias y cuales son compras más grandes/para largo plazo?
  - ¿Cuáles cosas puede construir que facilitaran el proceso?
  - ¿Cuáles actividades ayuda con este proceso/son necesarios que haga? ¿Cuáles oficios están relacionados?
- ¿Qué piensa usted es la parte más difícil de manejar/gestionar las necesidades de su y su familia de mantener la casa higiénica, incluso el manejo de heces? ¿Qué parte es lo más fácil?
  - ¿Qué tipo de inodoro tiene? ¿Dónde dispone de los contenidos? ¿Hay un sistema en la comunidad para tratar los desechos? ¿Cómo funciona cuando no hay agua?
  - ¿Que ayudara a usted a mantener una casa limpia, incluso el manejo de heces?
  - ¿Hay cosas en que no gasta dinero en este momento, pero que piensa que son importante para sus necesidades y compraría si podría?
  - ¿Cuáles cosas relacionadas al manejo de heces son compras diarias y cuales son compras más grandes/para largo plazo?
  - ¿Cuáles cosas puede construir que facilitaran el proceso?
  - ¿Cuáles actividades ayuda con este proceso/son necesarios que haga? ¿Cuáles oficios están relacionados?
- Si usted tuviera $1,000 dólares para gastar (para usted/su familia/casa) pero solo podría gastarlo en cosas que facilitan el proceso de obtener agua potable/de consumo, agua doméstica, en la maneja los desechos, y el mantenimiento de una casa/familia higiénica, ¿cómo lo gastaría? ¿Por qué?
- ¿Para comprar o construir una cisterna o un baño/inodoro nuevo, cuánto dinero necesitara tener disponible? ¿Porque o porque no quiere comprar o construir ese tipo de cosas?
- Siente que la comunidad o el lugar en que usted y su familia viven haga una diferencia en su habilidad de obtener agua potable/de consumo o agua doméstica o mantener de una casa higiénica, incluso el manejo de heces?
  - ¿Tiene un sistema alcantarillado en su comunidad? ¿Cómo funciona?
  - ¿Cómo si o cómo no? ¿Es más fácil o más difícil para madres en otras comunidades para obtener esas cosas? ¿Por qué?
  - ¿Qué le gustaría tener en su comunidad que no tiene ahora?
  - ¿Qué tiene una comunidad “ideal”?
- ¿Qué hace usted para evitar tener diarrea/evitar que sus niños y su familia tienen diarrea?
  - ¿Depende en cosas que puede comprar, o cuánto dinero tiene, o dónde vives?
  - ¿Algunas de las cosas de las actividades de los dos sorteos son importantes?
  - ¿Qué hacen madres en otras comunidades para evitar tener diarrea?
- ¿Qué hace usted para evitar el dengue/evitar que sus niños y su familia contagie del dengue?
  - ¿Depende en cosas que puede comprar, o cuánto dinero tiene, o dónde vives?
  - ¿Algunas de las cosas de las actividades de los dos sorteos son importantes?
  - ¿Qué hacen madres en otras comunidades para evitar tener el dengue?
