# Supplementary material for: Socioecological drivers of water, sanitation, and hygiene (WASH) choices: A qualitative analysis of maternal perspectives in northwest Ecuador
Source: PLOS Water. Author manuscript; Available in PMC 2026 Feb 28. (PMC12948185; doi:10.1371/journal.pwat.0000368)
Supplement: S2 Text: Original, untranslated Spanish language versions of quotes included in this manuscript, in order of mention [file NIHMS2143457-supplement-S2_Text__Original__untranslated_Spanish_language_versions_of_quotes_included_in_this_manuscript__in_order_of_mention.docx]

**S2 Text: Original, untranslated Spanish language versions of quotes included in this manuscript, in order of mention**

A veces en la ciudad si, a veces uno se queda sin agua hasta los tres, cuatro días, pero acá por lo menos que del río la tenemos todo, todo el tiempo. **HH 7010**

Pues no sé cómo le llamaría pero que el agua saliera más limpia, porque a veces el agua sale como, como café como, como agua del río, del río y sale sucia. **HH 2125**

Una agua limpia que uno salga y abra la manguera y se vea una agua limpia que no hay que guardar, ja, ja, ja, ja…..eso…eso sirve para el baño y uno quiere agua para beber, no, ji, ji, ji. **HH 3117**

Cuando llueve duro hay gente que recoge su agua de lluvia ya pues cuando no llueve mandan el agua potable, solo que esté dañado no mandan, ahí si está dañado uno va al río agarrar su agua o sino uno pide y va a cargar allá en el agua potable, aja, eso. **HH 3207**

Bueno para conseguir agua cuando llueve… se recoge el agua de lluvia ya, y cuando ya entonces se recoge el agua de lluvia hay tiempos que la lluvia no, no está lloviendo entonces a veces nos toca agarrar del río y poner a hervir o sino pues si se tiene comprar su botellón de agua le manda a comprar. **HH 7008**

La diferencia que la de allá es más tratada y la de acá es como que bueno en término de que a nosotros nos toca que nos, o sea la clase media o baja nos toca por ejemplo ya comprar una agua como para estar tomando y ellos utilizan la misma agua para todo porque el agua de ellos es más tratada, la de nosotros bueno si hablamos en términos de grifo la llave ya es cosa que no es tan, no, no, no utilizamos como para cocinar, para poder no, o sea ya tenemos una agua específica como para poder cocinar y otra como para hacer oficio o hacer la limpieza acá. **HH 2414**

Uno con la cisterna uno la llena si no hay agua ya tiene de a dónde sacar su agua no le hace falta. **HH 3207**

Realmente tendríamos que nosotros mismo tratarla he, ya ponerle su abate y estarlo he, por ejemplo limpiando consecutivamente porque también a veces viene de que cuando se pone la cisterna también viene de que adentro se, el agua como que se, se ha juntado algo así ya… Incluso hasta cucarachas se le meten ahí… Sapos… y a veces uno no se da cuenta y el agua está corriendo por toda la casa y uno piensa que el agua está siendo bien tratada y no es así, si, son he, siempre para uno mantener un tanque o algo tiene que estarlo revisando continuamente, lavando continuamente si para que pueda tener seguridad por su agua. **HH 2414**

Mejor sería que venga directamente el agua de la llave y no tener como cuando hay la cisterna porque hay veces que el agua de la cisterna pasa mucho tiempo y como que se abomba así entonces también hay que estarla limpiando, eso mantenerlo limpio también no es, no es bueno que el agua está así, mejor sería directamente el agua a la llave no ver la cisterna por allá. **HH 2125**

Aquí porque no es mi casa. **HH 3207**

Porque no hay un lugar adecuado donde se la pueda hacer, no hay un lugar adecuado. **HH 4012**

Porque el terreno no se presta para estar haciendo excavaciones para hacer porque abajo es pura peña, peña fina que no puede que el hacha rebota aja. **HH 4014**

Por ejemplo lo más barato como quién dice para he, talvez barato en corto plazo pero a largo plazo es costoso el bidón de agua porque por ejemplo usted puede talvez, porque a mí me dejan por mes, porque se me hace más fácil porque toca comprar todos los días, ha, entonces al mes me deja el señor entonces es mensualmente uno cobra y paga como a, por eso le vuelvo y le repito a corto plazo es económico pero a largo plazo es costoso porque si nos ponemos a calcular al año cuánto uno se gasta, pero es lo más accesible creo que es el bidón de agua, aja. **HH 3124**

Mire pue, ellos allá tienen más, más posibilidades de agua y todo acá uno no tiene, ellos allá tienen todo ya, y acá uno tiene que comprar y buscar todos los día, allá no, ellos allá no compran todos los días y lo que pagan es mensual mientras acá nos toca pagar todos los días, cuando se acaba el tanque de agua uno tiene que comprar. **HH 2237**

Ha, si tuviera si, ja, ja, si….como no querer. **HH 2031**

Porque uno de allá tiene que subir la poma y uno se cansa, a veces no llueve y no hay dinero para comprar los bidones, y es duro. **HH 11**

Difícil, difícil si porque se seca el, y si los que tienen su agua van a llenar tenemos que hacer llenar, lléname un tanque de agua y lo cobran a dólar y medio el tanque cuando no llueve, pero cuando llueve yo hay si con mi pierna así salgo allá afuera trato de llenar los tanque que tengo allá a las poma, los tacho, los balde, pero cuando no llueve entonces los que llenan con bomba a uno le, cada uno un tanque dólar y medio. **HH 7008**

Es que para tener la casa limpia y aseada tiene que haber es agua y yo no veo cuál es la diferencia si no hay, si no tiene agua pues por más que barra y si no puede asear el baño cómo hacemos. **HH 1046**

He, lo primordial sería el agua…..este, también uno tiene su desinfectante para limpiar el piso, he, tiene para mantener limpia las sillas todo para que esté todo desinfectado pero lo primordial para tener todo limpio es el agua. **HH 1149**

*Socioecological model quotes, listed from climatic to individual*

Difícil llenar agua porque hay que estar llena esperar que la gotera caiga y llene el envase para traspasar difícil…….llenar pomas, pero hay que hacerlo pues si ahí nos ahorramos los tres dólar que uno se ahorra, uno lava uno todo hace. **HH 2331**

Cuando se crece el río, que los río salen fuera de control se hunde pues, esto llega el agua hasta aquí entonces me imagino que se tapa y se llena de lodo. **HH 7008**

Esto es una invasión y uno a veces tiene que coger y pensar de qué manera lo hace, cómo lo hace porque igual esto no es de uno ya pero uno igual por su comodidad uno lo hace, lo trata de, de hacerlo, pero si diferencia igual si hay usted ve aquí calles no hay, en otras partes en, en otras partes si hay, hay uno se rebusca con lo que es el baño, con lo que es el agua nos rebuscamos tratamos pues en lo más posible de conseguir unas cosas que en otras partes si hay. **HH 2029**

Las familias ricas ellos…….ellos tienen su cisterna, su, su algiber en su hogar porque digamos en el tiempo de que no hay agua en la comunidad ellos tienen su respaldo porque tienen su algiber seguro. **HH 1149**

El agua pues yo no sé qué traten mejor está muy sucia huuu esa agua huuuu como para beber creo que le queda todo mugre en las tripa, ja, ja, ja, ja… **HH 3117**

Se compra el tanque de, aquí un tanque así cuesta tres dólar…. a veces no hay esos tres dólare … Que le hacemos …..nos aguantamos hasta que consigamos para pagarlo ya… Es difícil porque a veces no, no hay agua cuando uno necesita y hay que estar prestándola así para poder comprar el agua….aja, y ante eso es bastante duro porque de una u otra forma tiene que endeudarse en plata para el agua. **HH 2031**

Para mí es difícil porque a veces necesito de urgente y me quedo más con la bebe… tengo que dejar la puerta un poco ajustada ir rápido al río y subir porque ya para atender la bebe agarra el agua lo que voy a utilizar en la cocina y lo que voy a echar en el tacho del baño, ya. **HH 4014**

Lavadora también es importante porque igual aquí para yo poder lavar yo tengo que dejarlas a ellas sola y me bajo al pozo, lavo ahí en el pozo, cuando lleno el tanque me pongo a lavar aquí. **HH 2029**
