# Supplementary figures and images for: Socioecological drivers of water, sanitation, and hygiene (WASH) choices: A qualitative analysis of maternal perspectives in northwest Ecuador
Source: PLOS Water. Author manuscript; Available in PMC 2026 Feb 28. (PMC12948185; doi:10.1371/journal.pwat.0000368)

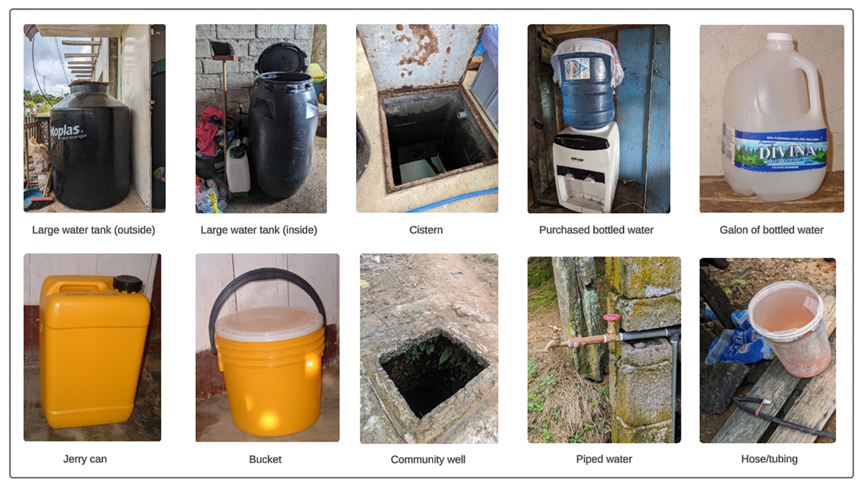

Supplement: S1 Fig. Types of household water storage containers or water access points frequently utilized in households in the study region. [file NIHMS2143457-supplement-S1_Fig__Types_of_household_water_storage_containers_or_water_access_points_frequently_utilized_in_households_in_the_study_region_.tif]
